# Atypical asymmetric lattice corneal dystrophy associated with a novel homozygous mutation (Val624Met) in the *TGFBI* gene

**Published:** 2008-03-12

**Authors:** Natalie A. Afshari, Rosanna P. Bahadur, David E. Eifrig, Ida B. Thogersen, Jan J. Enghild, Gordon K. Klintworth

**Affiliations:** 1 Department of Ophthalmology, Duke University Medical Center, Durham, North Carolina; 2 Department of Pathology, Duke University Medical Center, Durham, North Carolina; 3 Department of Molecular Biology, Aarhus University, Aarhus, Denmark

## Abstract

**Purpose:**

To evaluate the *TGFBI* gene and the encoded transforming growth factor beta-induced protein (TGFBIp) in a 47-year-old African-American patient with an unusual atypical asymmetric lattice corneal dystrophy (LCD).

**Methods:**

The eyes of the proband and his brother were examined by slit-lamp biomicroscopy and their clinical records were reviewed. All 17 exons of *TGFBI* were evaluated in genomic DNA extracted from blood or buccal epithelial cell samples from the proband and his family members. The corneal tissue of the proband was examined histopathologically, and TGFBIp was analyzed in half of an excised corneal button.

**Results:**

The proband (who had an unusual atypical asymmetric LCD) and his brother (who had mild bilateral deep stromal opacities) were found to have homozygous Val624Met mutations in *TFGBI*. The proband’s daughter who was heterozygous for the Val624Met mutation had no reported ophthalmic abnormalities. The corneal tissue from the proband contained TGFBIp with the Val624Met mutation. Patients with LCD have different clinical phenotypes based on their genotype. Molecular genetic analyses are becoming increasingly important in making precise diagnoses and prognostic predictions about inherited corneal disorders.

**Conclusions:**

A novel Val624Met homozygous mutation in *TGFBI* was associated with atypical LCD in two family members. Symptomatic corneal disease was absent at the age of 24 years in the offspring of the proband who was heterozygous for this mutation. This is an apparent example of a *TGFBI* mutation that becomes evident when it is homozygous. The finding of Val624Met mutated TGFBIp in a ~65 kDa protein band in a reduced sodium dodecyl sulfate (SDS) gel suggests that the accumulated protein was intact TGFBIp and not a fragment of TGFBIp.

## Introduction

The lattice corneal dystrophies (LCDs) are inherited diseases characterized by linear opacities composed of amyloid within the corneal stroma. The classic form of LCD (LCD type 1), which is inherited as an autosomal dominant trait, was originally described by Biber [1], Haab [2], and Dimmer [3] in the late 1800s. Fine refractile lines in a lattice meshwork appear in the anterior corneal stroma centrally and spread to the periphery. This type of LCD typically presents itself in the first decade of life with symptoms of recurrent corneal erosions and decreased vision.

In 1992, the *TGFBI* (*BIGH3*) gene was identified by Skonier et al. [4] in a study of genes induced by transforming growth factor-β in a human adenocarcinoma cell line. Several years later, Munier et al. [5] reported mutations in *TGFBI* in several inherited corneal disorders with different phenotypes including LCD type 1. Most frequently, LCD type I has been associated with an Arg124Cys mutation in exon 4 of *TGFBI* [5], but other mutations also cause this phenotype [6,7], other variants of LCD, or corneal amyloidosis. Abnormal accumulations of amyloid in corneas of affected patients are believed to contain either the transforming growth factor beta induced protein (TGFBIp) or its degradation products.

Here, we report a novel mutation in *TGFBI* that was found in a patient with an atypical asymmetric form of LCD.

## Methods

Duke University Institutional Review Board approval was obtained for this study, which adhered to the tenets of the Declaration of Helsinki.

Initially, a patient with unilateral linear corneal opacities was referred to Duke University Eye Center. The proband and his brother were evaluated clinically by slit-lamp biomicroscopy. Available clinical records, family history, and photographs were reviewed. The proband underwent a penetrating keratoplasty, and a histopathologic study was performed on the excised corneal button. This tissue specimen was sectioned and stained with hematoxylin and eosin, Masson trichrome, periodic acid Schiff, and Congo red. Transmission electron microscopy was not performed. Half of the proband’s cornea was frozen for protein analysis. This was done after the specimen was lyophilized, homogenized, and boiled in sample buffer at a concentration of 2 mg/ml [8]. The sample was reduced with dithiothreitol (DTT) and loaded on sodium dodecyl sulfate (SDS–PAGE) at 100 µg per lane. The band containing TGFBIp was excised and digested with trypsin. The released peptides were micropurified and subjected to mass spectrometry on a quadrupole time-of-flight instrument (Q-TOF Ultima Global, Micromass, Manchester, UK) equipped with both MALDI (matrix-assisted laser desorption ionization) and nanoelectrospray ion sources.

Buccal swabs were obtained from the proband’s 24-year-old daughter who reportedly had no ocular disease. DNA was extracted from venous blood or buccal swabs of all three subjects. DNA from blood was obtained using the Puregene™ Blood Kit (Gentra Systems, Minneapolis, MN) and from buccal swabs using the Puregene^TM^ buccal cell kit (Gentra Systems). Genomic DNA of all exons of *TGFBI* was amplified by the polymerase chain reaction (PCR) using forward and reverse primers as documented elsewhere [9]. The resulting PCR products were purified using QIAquick® PCR Purification Kit (Qiagen, Valencia, CA), sequenced on both strands using the Big Dye Terminator Cycle Sequencing system (Applied Biosystems, Foster City, CA), and combined with an ABI 377 Prism DNA Sequencing instrument (Applied Biosystems). The resulting DNA sequencing gel was analyzed using the ABI Prism DNA Sequence Analysis software program (Applied Biosystems). The sequences were then aligned to *TGFBI* cDNA using the SeqWeb, Sequence Analysis web-based program (Accelrys, San Diego, CA) to look for any nucleotide changes and amino acid changes in the sequences when compared to *TGFBI* cDNA. The entire coding region of *TGFBI* was sequenced with this technique as described elsewhere [6].

## Results

### Clinical findings

**Figure 1:**
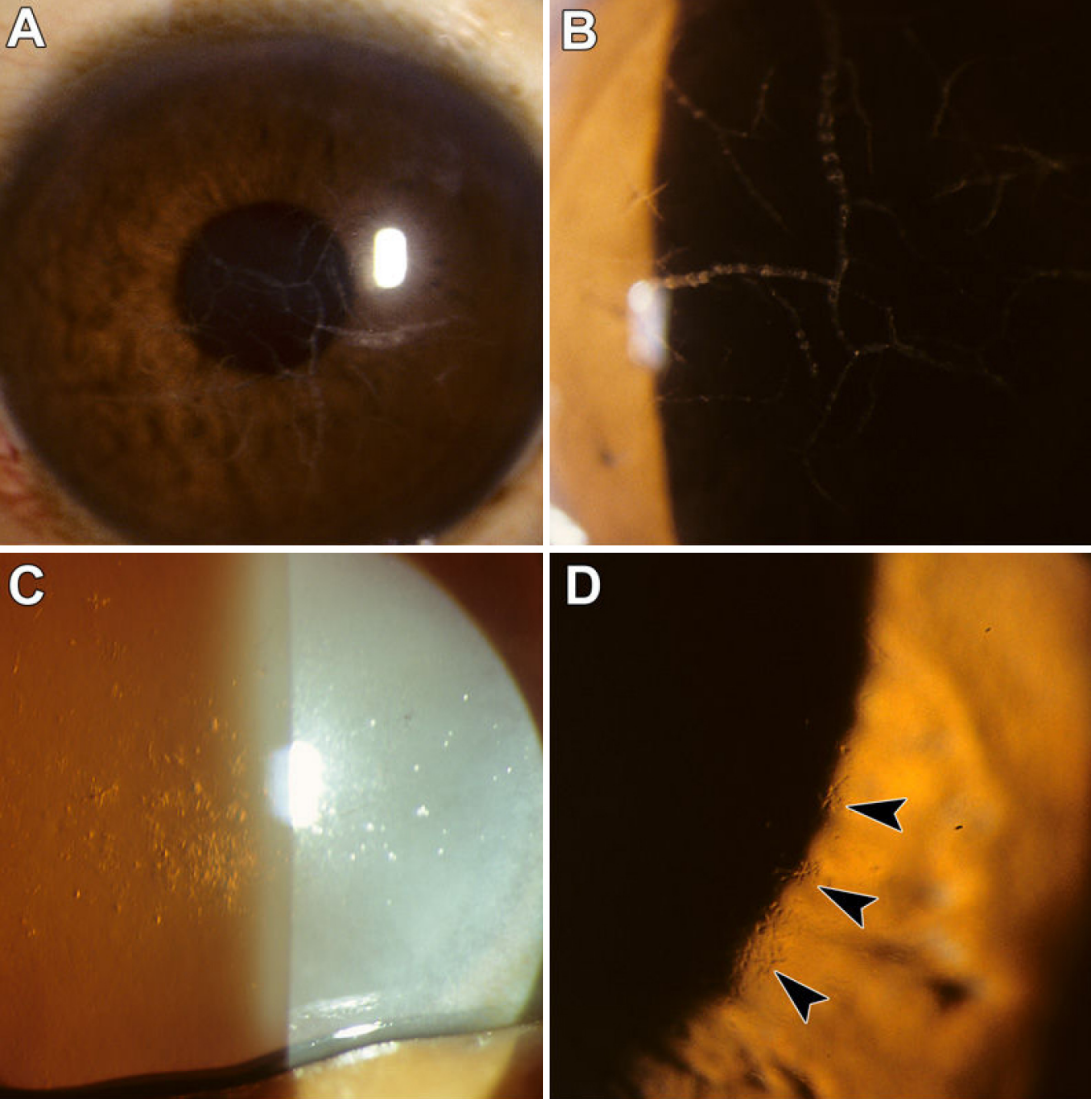
Slit-lamp photographs of the proband’s affected eye. **A** and **B**: Slit-lamp photographs of the ropy lattice lines in the proband’s affected eye at two different magnifications are shown. The proband was 47 years old. **C** and **D**: Slit lamp photograph of the right and the left eye of proband's brother showing multiple small geometric rod shaped lesions in the posterior corneal stromal (the lesions are highlighted with arrowheads).

The 47-year-old proband was hospitalized for corneal transplantation. He had noted decreased vision for several years before presentation; however, he had no prior documented eye examination. At the time of the penetrating keratoplasty, his best corrected visual acuity in the right eye was 20/30 and in the left eye was 20/50. Slit lamp biomicroscopy of the proband revealed unilateral thick, ropy linear opacities within the deep stroma of the left cornea (Figure 1A,B). His fellow eye contained small, deep, short rod shaped corneal stromal opacities, and both corneas of his brother (44 years of age) were found to have bilateral corneal opacities similar to those in the proband’s right cornea (Figure 1C,D). The proband’s 24-year-old daughter reported no ophthalmic symptoms by history and was unfortunately not available for a clinical examination of her eyes. The proband died of complications of chronic alcoholism at 49 years of age.

### Histopathology

Histopathologic evaluation of the excised corneal button revealed stromal deposits of amyloid, which was confirmed in Congo red stained preparations examined under polarized light. In keeping with the clinical impression, the amyloid was not located in the superficial corneal stroma but was found mainly in the mid-third of the corneal button.

### Genetic analysis

**Figure 2:**
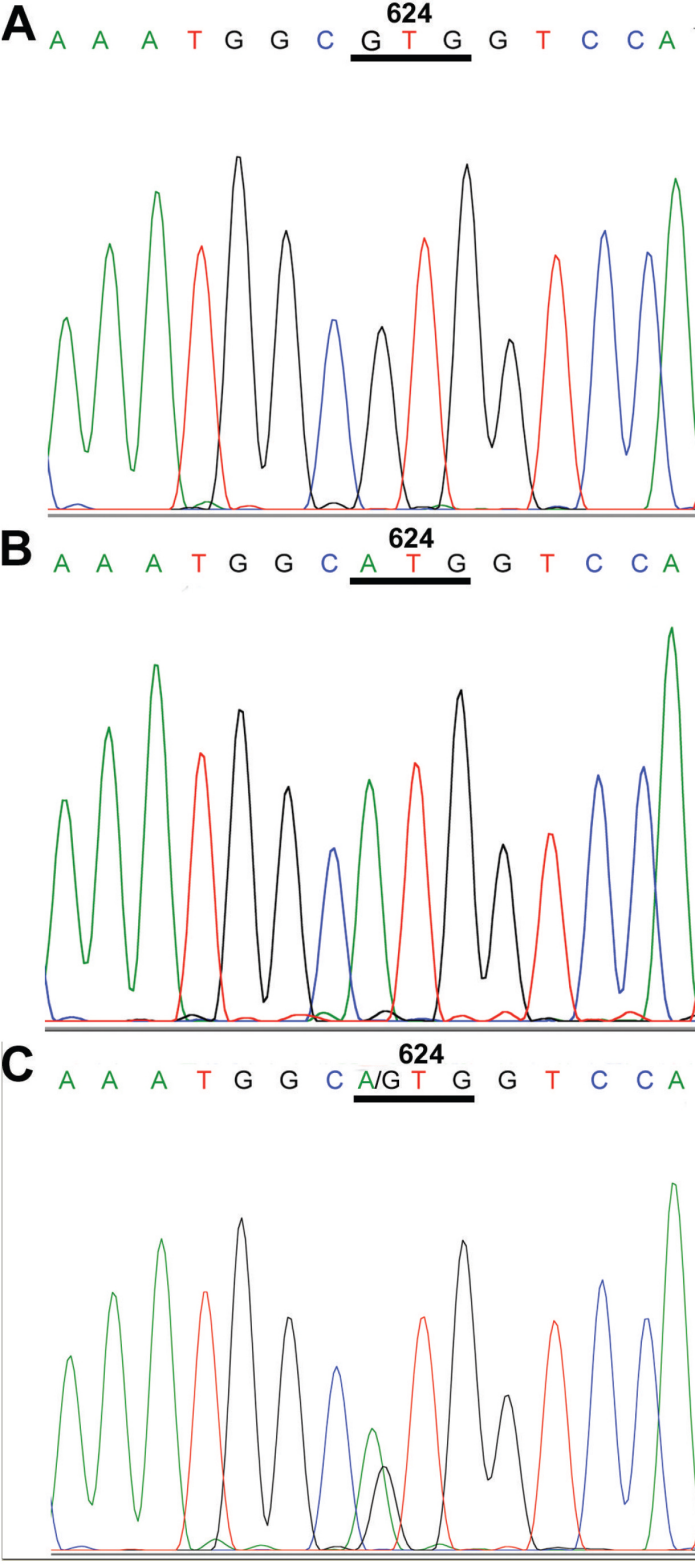
Partial nucleotide sequences of exon 14 of *TGFBI* in this family. **A**: A normal sequence is shown, which has valine in codon 624. **B**: The proband and his brother had homozygous substitutions in nucleotide 1870, which is predicted to alter the amino acid at codon 624 (Val624Met). **C**: The proband’s daughter had a heterozygous substitution of valine to methionine in the same codon. The observed nucleotide change in exon 14 of *TGFBI* has not been detected in the general population.

The proband had a homozygous nucleotide substitution of adenosine for guanine at nucleotide 1870 in *TGFBI* in exon 14, which led to a predicted novel amino acid change in codon 624 to methionine (Met) instead of valine (Val; Val624Met) shown in Figure 2. In addition, two single nucleotide polymorphisms, 981A>G (Val327Val) and 1620T>T (Phe540Phe), were also found in this individual. The proband’s brother also had the homozygous Val624Met substitution. As expected, the proband’s daughter had a heterozygous Val624Met change.

### Protein analysis

**Figure 3:**
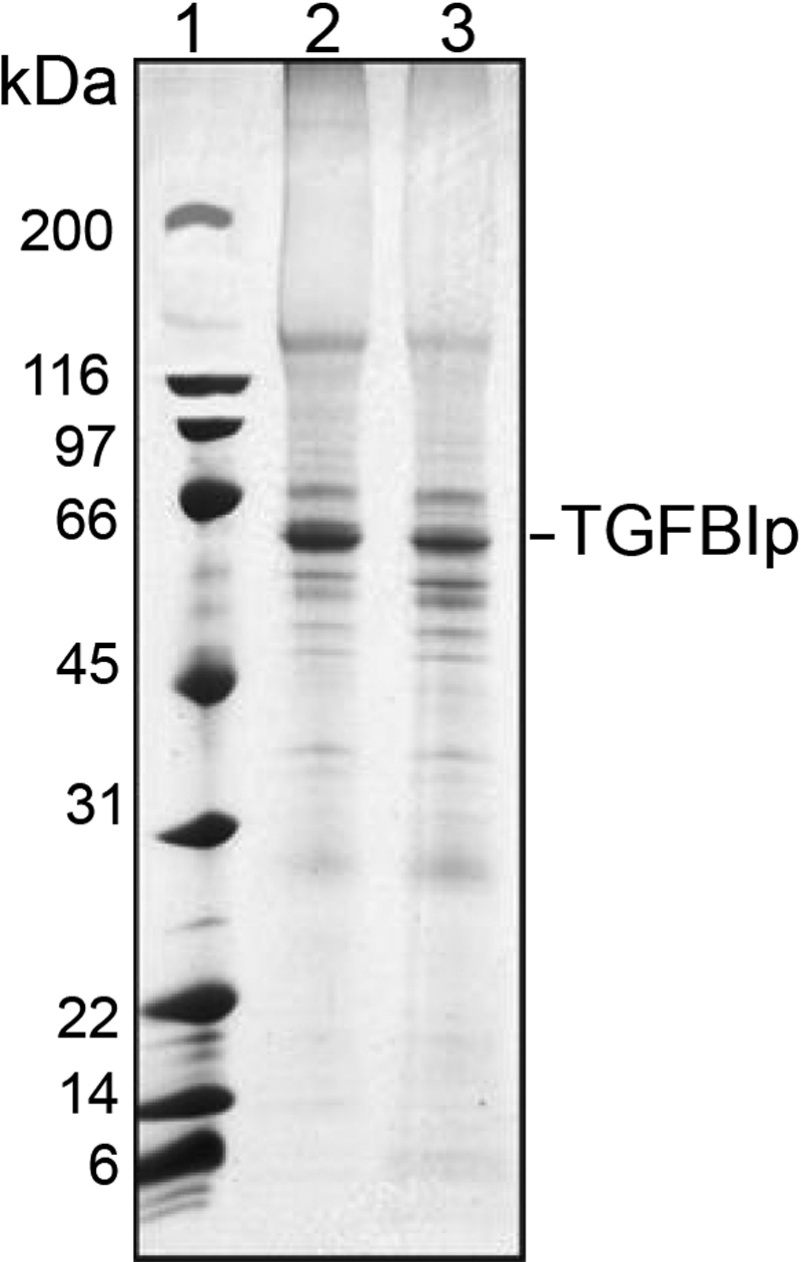
Comparison of SDS-extracted proteins derived from the proband and a normal human cornea. The extracted proteins were analyzed by reduced SDS–PAGE and stained with coomassie blue. The 64 kDa band in lanes 2 and 3 (see arrow) were excised, digested with trypsin, and identified as TGFBIp by MALDI mass spectrometry fingerprint analysis. Lane 1: molecular size markers; Lane 2: normal human cornea; Lane 3: cornea of proband.

The amount of soluble protein in the proband’s cornea was similar to that of a normal control cornea. The discernible individual bands in the SDS gels were also similar and of an equivalent intensity (Figure 3). In a normal human cornea, a peptide derived from the trypsin digest of TGFBIp contained Val-624 (3491.28 Da). In the proband’s cornea, this normal peptide was replaced with the mutated peptide Met-624 (3539.29 Da). The mass of the mutated peptide was 16 Da higher than expected because the methionine residue was oxidized. The tryptic peptide (Glu-611 – Arg-642) containing Val-624 was identified in the normal human cornea and the mass of the peptide was 3491.28 Da (MH^+^). In the proband’s cornea, the Glu-611 – Arg-642 peptide was identified with a mass of 3539.29 Da (MH^+^). This is consistent with a Val624Met mutation.

## Discussion

A novel homozygous change in *TGFBI* was found in two family members with atypical asymmetric LCD and deep corneal stromal opacities. The homozygous nucleotide change in exon 14 of *TGFBI* in this family not only coincided with an atypical LCD but predicts a valine to methionine change in the fas4 domain of the encoded TGFBIp, and this was indeed found on a direct analysis of the cornea. This homozygous nucleotide change is likely to be an important disease-producing mutation because it changes an amino acid residue in exon 14 from valine to methionine. Although both amino acid residues are hydrophobic, methionine contains a sulfur atom which is susceptible to oxidation and is slightly more bulky. These properties may affect the folding of the protein and hence its tertiary and quaternary structure. Perhaps a homozygous mutation is needed to affect the folding of the protein. This could explain why the 24-year-old heterozygous daughter of the proband lacks symptoms of corneal disease. Additionally, this region of exon 14 has been previously associated with other mutations leading to corneal dystrophies [7,10-12], thus a change in this exon is likely to be a disease-producing mutation. Stewart et al. [13] reported mutations on either side of this family’s mutation in codon 622 and 626 in three families with LCD. Only one of the reported mutations produced unilateral disease (His626Arg). The disorder was also late onset in most patients, and there was significant asymmetry.

Schmitt-Bernard et al. [12] reported the same His626Arg mutation in one family and a 9 bp insertion at position 1885 to position 1886 in exon 14 in another family with LCD.

Afshari et al. [10] also found the His626Arg mutation in a family with bilateral normal onset LCD and a Gly623Asp mutation in codon 14 in a family with Reis-Bücklers corneal dystrophy (granular corneal dystrophy type III). Furthermore, our reported mutation was not found in the 31 control samples from 27 other families in which mutations in the exon 14 of *TGFBI* gene were evaluated.

Patients with the different variants of LCD caused by mutations in *TGFBI* typically have bilateral, symmetric, translucent, delicate linear opacities that tend to be in the anterior stroma, and epithelial erosions often form. Unilateral LCD has been previously reported [14]. The linear and other shaped opacities result from amyloid deposits, but the reason for the amyloid accumulations remains poorly understood. In those corneal amyloidoses caused by mutations in *TGFBI*, it is clear that amyloid relates to mutations in specific codons.

Based on our findings in this family, the Val624Met mutation in *TGFBI* seems to also play a role in this regard. It is noteworthy that in addition to defects in *TGFBI*, corneal amyloidoses also results from genetic defects in at least two other genes (*GSN*, *TACSTD2* [formerly called *M1S1*]) that encode for different proteins [15,16].

Examining the mutated proteins encoded by the different genes is potentially helpful in understanding the role that the defect plays in altering the structure and function of the protein. In this case, the representative portion of cornea from the proband contained normal quantities of TGFBIp and other soluble proteins, but the analysis of the tryptic digest of TGFBIp in the proband’s cornea disclosed the predicted Val624Met mutation. Hence, these data show that the Val624Met TGFBIp mutant accumulated in the cornea. In addition, since the peptide was recovered from a ~65 kDa protein band in a reduced SDS gel, the observation suggests that the accumulated protein was intact TGFBIp and not a fragment of TGFBIp.

Heterozygous mutations in *TGFBI* have typically caused corneal dystrophies. However, in the family reported here, the novel Val624Met mutation apparently needs to be in a homozygous state for clinical disease to become evident as noted in the proband and his brother. Moreover, the proband’s 24-year-old daughter exhibited a heterozygous Val624Met al.teration in *TGFBI* but had no reported ophthalmic abnormalities. A comparable situation has been observed in a variation of LCD documented as LCD type III [17,18]. This late onset variant of LCD was thought to have an autosomal recessive mode of inheritance because it affected children of parents without corneal disease [17], but subsequently it became established that affected persons had a homozygous Leu527Arg mutation in *TGFBI* [19]. The Leu527Arg mutation, however, may be expressed clinically in the heterozygous state [20]. Future studies on other families with LCD will hopefully determine whether the Val624Met mutation is significant in the pathogenesis of LCD and whether a homozygous mutation is essential for disease expression.
